# Modulation of mitochondrial function with near-infrared light reduces brain injury in a translational model of cardiac arrest

**DOI:** 10.1186/s13054-023-04745-7

**Published:** 2023-12-14

**Authors:** Joseph M. Wider, Erin Gruley, Paul T. Morse, Junmei Wan, Icksoo Lee, Anthony R. Anzell, Garrett M. Fogo, Jennifer Mathieu, Gerald Hish, Brian O’Neil, Robert W. Neumar, Karin Przyklenk, Maik Hüttemann, Thomas H. Sanderson

**Affiliations:** 1 Department of Emergency Medicine, University of Michigan, 1500 East Medical Center Drive, Ann Arbor, MI 48109-5014 USA; 2 Max Harry Weil Institute for Critical Care Research and Innovation, University of Michigan, B10-103A, NCRC 2800 Plymouth Road, Ann Arbor, MI 48109 USA; 3 Department of Molecular and Integrative Physiology, University of Michigan, 7744 MS II, 1137 E. Catherine St., Ann Arbor, MI 48109-5622 USA; 4 Center for Molecular Medicine and Genetics, Wayne State University, 3214 Scott Hall, 540 E. Canfield Ave., Detroit, MI 48201 USA; 5 College of Medicine, Dankook University, Cheonan-Si, Chungcheongnam-Do 31116 Republic of Korea; 6 Department of Human Genetics, University of Pittsburgh, 130 De Soto Street, Pittsburgh, PA 15261 USA; 7 Neuroscience Graduate Program, University of Michigan, 204 Washtenaw Ave, Ann Arbor, MI 48109 USA; 8 Unit for Laboratory Animal Medicine, University of Michigan, North Campus Research Complex, 2800 Plymouth Rd, Ann Arbor, MI 48109 USA; 9 Department of Emergency Medicine, Wayne State University, 4201 St. Antoine St., University Health Center - 6G, Detroit, MI 48201 USA; 10 Clinical Research Institute, Children’s Hospital of Michigan, 3901 Beaubien Blvd, Detroit, MI USA; 11 Department of Pediatrics, Central Michigan University, 1280 S. East Campus Drive, Mount Pleasant, MI 48859 USA

**Keywords:** Cardiac arrest, Ischemia–reperfusion injury, Mitochondria, Oxidative phosphorylation, Near-infrared light, Mitophagy, Reactive oxygen species

## Abstract

**Background:**

Brain injury is a leading cause of morbidity and mortality in patients resuscitated from cardiac arrest. Mitochondrial dysfunction contributes to brain injury following cardiac arrest; therefore, therapies that limit mitochondrial dysfunction have the potential to improve neurological outcomes. Generation of reactive oxygen species (ROS) during ischemia–reperfusion injury in the brain is a critical component of mitochondrial injury and is dependent on hyperactivation of mitochondria following resuscitation. Our previous studies have provided evidence that modulating mitochondrial function with specific near-infrared light (NIR) wavelengths can reduce post-ischemic mitochondrial hyperactivity, thereby reducing brain injury during reperfusion in multiple small animal models.

**Methods:**

Isolated porcine brain cytochrome *c* oxidase (COX) was used to investigate the mechanism of NIR-induced mitochondrial modulation. Cultured primary neurons from mice expressing mitoQC were utilized to explore the mitochondrial mechanisms related to protection with NIR following ischemia–reperfusion. Anesthetized pigs were used to optimize the delivery of NIR to the brain by measuring the penetration depth of NIR to deep brain structures and tissue heating. Finally, a model of out-of-hospital cardiac arrest with CPR in adult pigs was used to evaluate the translational potential of NIR as a noninvasive therapeutic approach to protect the brain after resuscitation.

**Results:**

Molecular evaluation of enzyme activity during NIR irradiation demonstrated COX function was reduced in an intensity-dependent manner with a threshold of enzyme inhibition leading to a moderate reduction in activity without complete inhibition. Mechanistic interrogation in neurons demonstrated that mitochondrial swelling and upregulation of mitophagy were reduced with NIR treatment. NIR therapy in large animals is feasible, as NIR penetrates deep into the brain without substantial tissue heating. In a translational porcine model of CA/CPR, transcranial NIR treatment for two hours at the onset of return of spontaneous circulation (ROSC) demonstrated significantly improved neurological deficit scores and reduced histologic evidence of brain injury after resuscitation from cardiac arrest.

**Conclusions:**

NIR modulates mitochondrial function which improves mitochondrial dynamics and quality control following ischemia/reperfusion. Noninvasive modulation of mitochondria, achieved by transcranial treatment of the brain with NIR, mitigates post-cardiac arrest brain injury and improves neurologic functional outcomes.

**Graphical abstract:**

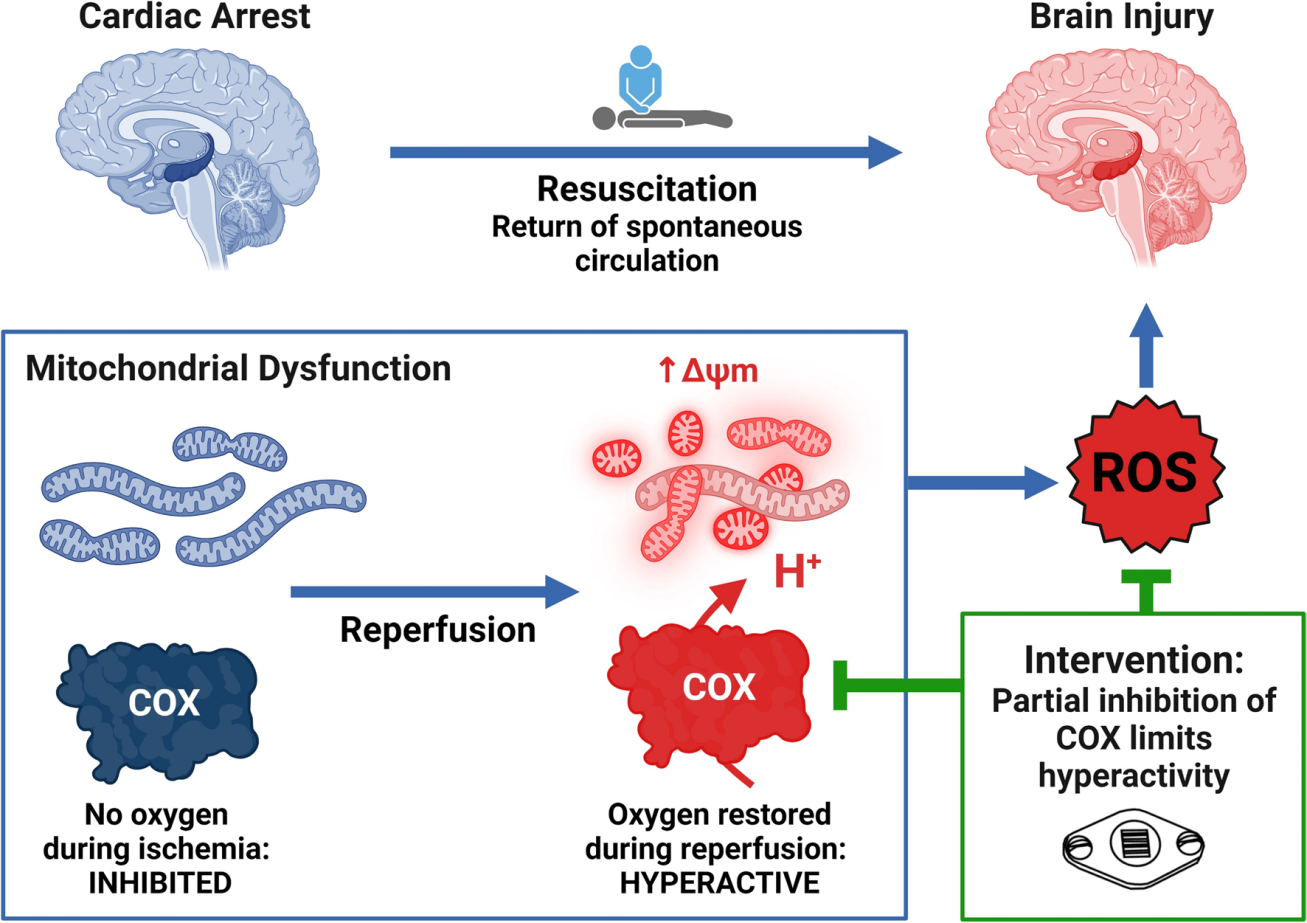

**Supplementary Information:**

The online version contains supplementary material available at 10.1186/s13054-023-04745-7.

## Background

Ischemia–reperfusion (I/R)-induced brain injury is a primary cause of poor clinical outcomes associated with cardiac arrest and resuscitation [1]. The survival rate for out-of-hospital cardiac arrests is < 10%, and in-hospital mortality is 70% [2–4]. Neurological recovery remains poor in survivors of cardiac arrest, with cognitive deficits reported in 50% of survivors at 6 months and 25% at 4 years [5, 6]. Although a significant amount of preclinical evidence has demonstrated that I/R injury is a gradual process and the development of post-resuscitation brain injury can be interrupted, translational efforts have been largely unsuccessful. Neuroprotective options remain limited despite the clear demand for strategies that reduce I/R injury.

**Fig. 1:**
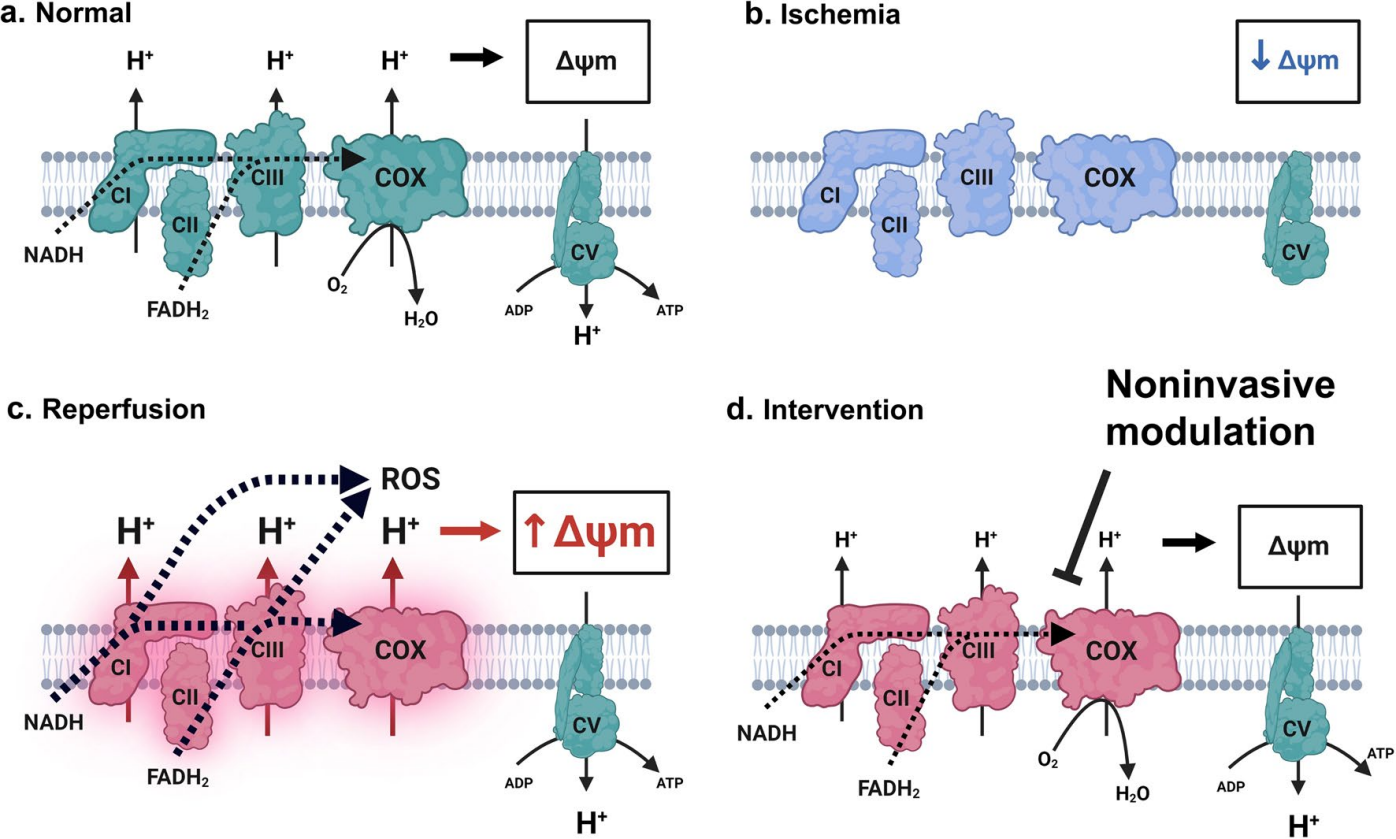
Mechanism of ischemia–reperfusion injury and NIR-mediated neuroprotection. **A** Under normal conditions, complex I (CI) and complex II (CII) transfer electrons (from NADH and FADH_2_, respectively) through complex III (CIII) to cytochrome *c* oxidase (COX), where oxygen is reduced to water. Energy from the electron transfer is used to pump protons from the matrix into the intermembrane space, generating the mitochondrial membrane potential (ΔΨ_m_). Complex V (CV) then uses this electrochemical gradient to generate ATP. **B** During ischemia, oxygen and electron donors are depleted and the ΔΨ_m_ collapses. Regulatory mechanisms function to maintain the ΔΨ_m_ by activating the respiratory complexes. Without oxygen, the ΔΨ_m_ cannot be restored resulting in a hyperactivated, yet inactive, respiratory chain. **C** During reperfusion, oxygen allows respiration to resume and increased activity of the respiratory chain during reperfusion leads to hyperpolarization of the ΔΨ_m_ and reactive oxygen species (ROS) generation. **D** Intervention by noninvasive modulation of COX limits hyperactivity of the respiratory chain and prevents hyperpolarization of the ΔΨ_m_. This allows the respiratory chain to gradually return to a normal activation state without hyperpolarizing the ΔΨ_m_. Figure generated with BioRender.com

Production of mitochondrial reactive oxygen species (ROS) is a fundamental injury mechanism driving the early progression of I/R injury [7–10]. However, drugs designed to scavenge ROS generated during reperfusion have yet to result in a clinically effective therapy. The generation of mitochondrial ROS in the brain is dependent on the mitochondrial membrane potential (ΔΨ_m_), an electrochemical gradient across the inner membrane that powers ATP synthesis. In healthy mitochondria, respiration is highly regulated through cell signaling cascades, allosteric control and feedback mechanisms that optimize ΔΨ_m_ to meet cellular energy demand while minimizing ROS production (Fig. 1A) [11, 12]. We and others have demonstrated that hyperpolarization of ΔΨ_m_ is a driver of ROS generation during reperfusion [13–21]. Under circumstances of high energy demand, regulatory mechanisms favor respiration to increase ATP availability. For example, increased cellular activity, succinate accumulation, ATP depletion, ADP accumulation and ion imbalance activate respiration in neurons; however, during ischemia oxygen is not present, precluding mitochondrial respiration (Fig. 1B) [22]. In contrast to healthy conditions, cellular stress induced by ischemia favors stimulatory mechanisms that override inhibitory signals, causing hyperpolarization of ΔΨ_m_ and an exponential increase in ROS generation (Fig. 1C) [11, 15, 18–21, 23, 24]. Accordingly, mitigating hyperactivation of respiration and preventing ΔΨ_m_ hyperpolarization would prevent ROS generation and limit the progression of reperfusion injury (Fig. 1D).[25].

NIR has been evaluated as a treatment for I/R injury in multiple tissues, including myocardium [26–29], forebrain [30–34] and skeletal muscle [35, 36]. The traditional mechanistic theory purports that NIR evokes a salutary biological effect by increasing ATP synthesis through stimulated mitochondrial respiration [37, 38]. Our group has recently confirmed that specific wavelengths in the NIR spectrum stimulate mitochondrial respiration through directly increasing cytochrome *c* oxidase (COX) activity, including the frequently studied 808 nm range. However, in contrast to previous studies on NIR, our studies uncovered a novel inhibitory effect of two wavelength ranges of NIR, 750 nm and 950 nm, that partially inhibit COX activity and consequently reduce mitochondrial respiration [39]. We have postulated that attenuating COX activity with NIR will prevent hyperactivation of respiration and limit ΔΨ_m_ hyperpolarization during the early stages of reperfusion [40]. Our studies in a cell model of ischemia/reperfusion injury and a rodent model of global brain ischemia showed that inhibitory wavelengths of NIR provide robust neuroprotection, whereas activating NIR (810 nm) was not protective [39]. In further support of the concept that inhibitory NIR reduces oxidative stress, inhibitory wavelengths were found to reduce ROS generation caused by I/R injury in vivo. The aim of the present study was to evaluate the mechanisms, neuroprotective efficacy, feasibility and safety of NIR therapy in a clinically relevant, translational model of cardiac arrest and resuscitation.

## Methods

### Cytochrome *c* oxidase activity

This study was approved by the Institutional Animal Care and Use Committees of the University of Michigan and Wayne State University and was performed in accordance with the Guide for the Care and Use of Laboratory Animals from the Institute of Laboratory Animal Resources. For oxygen consumption and ATP level measurements, porcine forebrain tissue was isolated from a naïve animal and subjected to a period of incubation at 37 °C to simulate ischemia. COX was isolated using our protocol optimized for maintaining post-translational modifications and thus regulatory properties of the enzyme [42]. In brief, control or ischemic porcine brain was homogenized, and the tissue extracts (*n* = 6 control, *n* = 8 ischemic) were loaded onto a DEAE-Sepharose anion exchange column (GE Healthcare) equilibrated with running buffer (125 mM phosphate buffer and 0.5% Triton X-100, pH 7.4). COX was eluted from the column via gradient elution with 100–700 mM phosphate buffer with 0.1%Triton X-100, pH 7.4. Ammonium sulfate precipitation was then performed to yield purified COX enzyme. Dialysis of purified COX enzyme was performed in 1 L solubilization buffer using a 12–14 kD Spectra/Por 2 dialysis membrane (#08700–150, Spectrum Laboratories, Inc.; Rancho Dominguez, CA, USA) overnight at 4 °C in order to remove cholate, which inhibits COX enzyme activity [43]. Oxygen consumption rate (OCR) of COX was analyzed in a light-protected Clark-type oxygen electrode chamber (DW2/2 chamber, Oxygraph system, Hansatech) in the presence of 15 nM of isolated COX, 20 mM ascorbate, 4 µM cytochrome *c* in OCR buffer containing 10 mM K-HEPES (pH 7.4), 40 mM KCl and 1% Tween 20. OCR was determined in the absence or presence of 750 nm and 950 nm NIR at the indicated power density (mW/cm^2^) values at 25 °C. OCR values were corrected for baseline, NIR-untreated values. Data were recorded and analyzed using Oxytrace + v1.0.48 software (Hansatech). COX activity is reported as % of control or nmol O_2_/min/mg protein. The experimental protocol is described in detail in reference [39].

### ATP Levels

As previously described [41], tissue samples from control or ischemic porcine brains (see below) were flash frozen in liquid nitrogen and stored at − 80 °C. Forebrain tissue samples (N = 6 control, N = 8 ischemic) were rapidly lysed via boiling for 2 min in 300 μL boiling buffer (100 mM Tris–Cl, 4 mM EDTA, pH 7.75) and sonicated on ice. Samples were diluted 300-fold and 40 μL of the diluted samples were used to determine ATP concentration with the ATP bioluminescence assay kit HS II (#11,699,709,001, Roche) according to the manufacturer’s protocol on an Optocomp I luminometer (MGM Instruments). Total protein of each sample was measured using the DC protein assay kit (#5,000,111, BioRad). The ATP level is reported as nmol ATP/g protein.

### Mitochondrial analysis in primary neurons

Primary cortical neurons were extracted from postnatal day 0–2 (P0-P2) mouse pups and seeded separately for individual biological replicates as previously described [44]. Cells were seeded at 300,000 cells/cm^2^ and incubated at 37 °C in 5% CO_2_. Half-media changes occurred every 3–4 days with neurobasal complete medium until oxygen–glucose deprivation (OGD) experiments at day-in-vitro 14. Simulated ischemia with OGD was achieved utilizing an O_2_ Control InVitro Glove Box (Coy Lab Products). The hypoxic chamber was maintained at 0.1% O_2_ and 5% CO_2_. OGD media, composed of 0.20 g/L CaCl_2_ (Spectrum, CA138500GM), 0.4 g/L KCl (Fisher Chemical, P217-500), 0.097 g/L MgSO_4_ (Fisher Chemical, M65-500), 6.8 g/L NaCl (Fisher Chemical, S2711), 2.2 NaHCO_3_ (Acros Organics, AC447102500), 0.14 g/L NaH_2_PO_4_-H_2_O (Fisher Chemical, S369-500 and 0.01 g/L Phenol red (Fisher Chemical, P74-10), was bubbled with 95% N_2_/5% CO_2_ inside of the hypoxic chamber for 60 min. Cells were transferred into the hypoxic chamber, washed twice with OGD media and then incubated with OGD media inside the hypoxic chamber for 150 min. After OGD, cells were removed from the hypoxic chamber, and media was replaced with Neurobasal medium without antioxidants (complete neurobasal medium with B27-AO (Gibco, 10,889,038) and incubated at 37 °C in 5% CO_2_ for various times of reoxygenation. NIR treatment was administered at the onset of reoxygenation for 2 h. Cells were treated with LED array chips (Roithner Lasertechnik, Vienna, Austria. One LED emitting 750 nm at 14 mW/cm^2^ and one emitting 950 nm at 14 mW/cm^2^). Cells were subsequently fixed with 4% paraformaldehyde and coverslipped for microscopy (Fig. 3A).

### Mitochondrial imaging and mitophagy analysis

Mitochondrial morphology and mitophagy, including characterization of mitochondrial morphology, were quantified in primary neurons, as previously described [45]. Neurons were extracted from MitoQC mice, which express a GFP-mCherry tag targeted to the outer mitochondrial membrane. Upon transport into the lysosome, the GFP is degraded by the low pH, yielding red mCherry fluorescence and allowing visualization of mitophagy flux. Increased mitophagy results in decreased GFP, while reduced mitophagy increases GFP signal. GFP was used as a mitochondrial marker for morphological categorization. We analyzed mitochondrial morphology with our custom machine learning-based morphologic analysis tool using a trainable Weka segmentation classifier model and machine learning-based classification of mitochondrial objects with the R Caret package and a trained random forest algorithm [44, 45]. Morphologic descriptors in this model have been validated with loss of function of mitochondrial dynamics and mitochondrial permeability transition pore opening (i.e., Drp1, Opa1 and CypD) [45]. Twelve random images were acquired for each biological replicate utilizing a Zeiss Axio Observer.Z1 inverted microscope at 63X oil immersion. Z-stacks were acquired spanning the mitochondrial network and processed using the Zeiss Zen Pro extended depth of focus wavelets algorithm. Each image was deconvolved using the regularized inverse filter method on Zeiss Zen Pro software. Images were further processed in Fiji ImageJ to segment and count mCherry-only puncta and were then batch processed as described in [44, 46].

### NIR tissue penetration and safety

Light penetration was determined with acute surgical procedures in a subgroup of pigs, independent from the efficacy experiments detailed below. The NIR light source was placed at the surface of the scalp and a light sensor (Thor Labs PM160 Power Meter) was inserted into the brain at the area of the hippocampus through a surgical craniotomy (Fig. 4B), and positioning was verified postmortem. Light attenuation was determined by analyzing the light levels through the pig scalp/skull/brain to the hippocampus in 3 independent instrumented animals (4 cm, *n* = 3) compared to the light levels with no subject between sensor and diode in 4 independent experimental frameworks (unimpeded–4 cm, n = 4). Heating was determined by surgically implanting thermocouples in the brain under anesthesia. Thermocouples (< 1 mm diameter) were placed on the surface of the cortex (cortex), in the hippocampus and in the skin.

### Surgical preparation: porcine model of cardiac arrest and resuscitation

Sample sizes were calculated based on experience with pilot studies and power analysis. Yorkshire-Hampshire pigs (3–4 months of age, 15 male and 15 female, 37.7 kg ± 4.6 kg) were induced with combined ketamine (33 mg/kg) and midazolam (1 mg/kg), and anesthesia was maintained by mechanical ventilation with isoflurane (1–4%). Ventilation was controlled to maintain normocapnia (35–45 mmHg). Body temperature was maintained at 38 °C ± 0.5 and SpO_2_, EtCO_2_, heart rate and ECG were monitored until the end of anesthesia (Table 1). Intravascular catheters were placed in the femoral artery and vein, and a pacing electrode was placed into the right ventricle through the jugular vein. Arterial blood samples were taken (baseline and 30 min post-resuscitation) for analysis of blood gases and lactate by i-STAT (Abbott Laboratories). Intravenous lactated Ringer’s solution at 200 mL/hr was continually administered throughout surgery. After cardiac arrest and resuscitation, the femoral venous line was tunneled to a subcutaneous port on the back for postoperative intravenous access.

**Table 1:** Experimental variables

		Sham	Cardiac arrest	Cardiac arrest + NIR
pH	Baseline	7.47 ± 0.04	7.49 ± 0.05	7.49 ± 0.05 (NS)
	30 min	7.48 ± 0.05	7.29 ± 0.09	7.33 ± 0.10 (NS)
PCO_2_ (mmHg)	Baseline	35.1 ± 4.10	37.15 ± 4.20	36.5 ± 4.20 (NS)
	30 min	36.28 ± 4.30	39.34 ± 12.80	42.65 ± 2.60 (NS)
PO_2_ (mmHg)	Baseline	362.6 ± 65.10	383.6 ± 72.20	394.9 ± 80.20 (NS)
	30 min	358.2 ± 26.60	318.0 ± 103.30	406.3 ± 53.90 (NS)
HCO_3_^-^ (mmol/L)	Baseline	25.67 ± 4.60	28.3 ± 3.40	28.44 ± 6.60 (NS)
	30 min	26.75 ± 2.50	21.0 ± 6.40	22.4 ± 4.00 (NS)
Lactate (mmol/L)	Baseline	0.862 ± 0.20	0.9 ± 0.55	0.68 ± 0.30 (NS)
	30 min	0.91 ± 0.30	4.97 ± 2.00	5.55 ± 1.30 (NS)
CPR etCO_2_ (mmHg)			18.20 ± 7.63	25.71 ± 8.81 (NS)
Time to ROSC (min)			12.14 ± 1.40	11.84 ± 1.00 (NS)

Blood gas parameters measured from fresh arterial blood with an i-STAT handheld blood analyzer at baseline (before arrest) and at 30 post-ROSC. Comparison of experimental variables was made with one-way ANOVA, and stepwise multiple comparisons were made between groups with Newman–Keuls post hoc test. Changes in pH, HCO_3_ (bicarbonate) and Lactate were observed between baseline and 30 min post-ROSC within groups, as compared by one-way ANOVA repeated measures with mixed effects. Values are reported as mean ± SD. Comparisons were made between cardiac arrest and cardiac arrest + NIR at each time point and *p* < 0.05 was considered significant. *NS* Not significant

### Cardiac arrest and resuscitation

Cardiac arrest (no-pulse, ventricular fibrillation with MAP of < 20 mmHg) was induced by utilizing a pacing electrode to apply direct current to the surface of the ventricular endocardium. Ventricular fibrillation was maintained for 8 min without mechanical ventilation or other intervention. To simulate bystander CPR, manual chest compressions were administered at 100/min (not goal-oriented) and were continued until ROSC. To simulate a delayed first responder arrival, defibrillation (biphasic, 100–120 J) was administered through ECG patches with a HeartStart MRx (Phillips Healthcare, Cambridge MA) beginning 2 min after the onset of CPR and attempted every 30 s thereafter until ROSC was achieved. Intravenous epinephrine (15–30 μg/kg) was also administered immediately after the first three defibrillation attempts (Fig. 5A).

### NIR treatment and recovery

The NIR device used for large animal cardiac arrest experiments was an alpha-prototype (Fig. 4A) that consisted of 10 wavelength-specific LED arrays, as described above (5 arrays emitting 750 nm and 5 emitting 950 nm). The LED arrays were secured to an aluminum heat sink and electrical fan for heat dissipation. Diodes were calibrated with an optical power meter (842-PE; Newport, Irvine, California, USA) and operated at an energy density of 200 mW/cm^2^ from each diode during therapy. Total surface area of the LED therapy device was 56 cm^2^.

This study considered sex as a biological variable by enrolling both male and female subjects (15 male/15 female). Before surgery, animals were randomly assigned to receive sham surgery (sham, n = 10; 6 male/4 female), cardiac arrest and resuscitation without NIR treatment (CA/CPR, n = 11; 4 male/7 female) and cardiac arrest and resuscitation with NIR treatment for 2 h (CA/CPR + NIR, n = 9; 5 male/4 female). Two pigs did not achieve ROSC and were not enrolled. Two pigs died post-ROSC in the CA/CPR group (1 male/ 1 female); therefore, histological endpoints in the CA/CPR group represent n = 9 that reached the study endpoint. Regardless of group assignment, surgical preparation, anesthesia duration and postoperative analgesics were consistent. For clinical relevance, NIR treatment was applied to the intact scalp. Topical glycerol was applied to improve transdermal light delivery and the diodes were positioned at a distance of 0.5 cm from the scalp. Following surgery, animals were sedated with diazepam (5 mg/mL, 1 mg/kg/hr) infused with lactated Ringer’s solution (LRS) (50 mL/kg/24 h) continuously for 18 h and buprenorphine (0.01–0.05 µg/kg) every 8–12 h. Animals were continuously monitored by laboratory personnel to manage temperature and sedation until fully recovered (~ 30 h). Neurologic deficit scores (NDS) were assessed by a blinded investigator. NDS grades the functional recovery based on a weighted scoring system from 0 (no deficit) to 150 (maximum deficit) that summates of scores for deficits in: consciousness, brainstem function, sensory responses, motor function, mobility, postural reflexes, spatial orientation and activity (Additional File 2: Table S1).

### Tissue harvesting and processing

Four days post-ROSC, the brain was flushed with 4 °C PBS and perfused with 4% paraformaldehyde (PFA) following euthanasia. Tissue was immersion fixed in 4% PFA for 48 h, cryoprotected in 30% sucrose and snap frozen. The entire hippocampus was cryosectioned at 60 μm in the coronal plane and every section mounted onto microscope slides. Hippocampal neurons stained with cresyl violet were quantified on a Zeiss inverted microscope with a 3-axis motorized stage to acquire data from three-dimensional tissue structures. Stereologic assessment of neuronal number in the CA1 and CA3 hippocampus was determined by an investigator blinded to experimental group using the optical fractionator probe in Stereo Investigator Software (MBF BioScience. Williston, VT) [47]. The CA1/CA3 region of the hippocampus was traced in 5–7 evenly spaced (240 μm apart) brain slices at low magnification (2.5×, Additional file 1: Fig. S1). The CA1 and CA3 regions were traced in a consistent manner between animals, determined by the morphology of the cells and their relationship to the subiculum and CA4 regions, respectively. The region of interest tracings were defined as the area of the hippocampus that included the highest density of cells within the defined CA regions. The region of interest was determined at high magnification (63x), looking specifically at cell morphology and location to determine appropriate CA regions, the software identified random counting frames within the traced region of interest (ROI) and subsequently recorded tissue thickness and total neuron count. In each tracing, 10% of the area was analyzed to ensure robustness of the stereological estimation and maintain the coefficient of error value under 0.10. Neurons were defined as (a) having the entire cell body within the thickness of the tissue section (i.e. without having been transectioned during cryosectioning) including 5 μm guard zones, (b) by size and (c) having an intact nucleolus. Neuron counts were normalized to tissue volume, estimated by the Cavalieri method using the tissue thickness and the area of the ROIs.

### Statistics

Population distribution was analyzed with Kolmogorov–Smirnov Test for normality. Data are reported as mean ± standard deviation (SD). Groups were compared using a two-tailed Student t test with two groups or ANOVA followed by Newman–Keuls multiple comparisons test for post hoc analysis where there were more than two independent groups. Data comparing multiple groups over time were evaluated with repeated measures ANOVA followed by Dunnett’s multiple comparisons test if group numbers were equal or mixed effects analysis with repeated measures, multiple comparisons with Sidak’s test for unequal group sizes. Statistics were analyzed and visualized using GraphPad Prism® version 9 (GraphPad Inc, La Jolla, CA, USA) and artwork and figures were assembled with Adobe Illustrator v.24.0.1 (Adobe Inc, 345 Park Ave, San Jose, CA) and with BioRender.com (49 Spadina Ave, Suite 200 Toronto, ON).

## Results

### Modulation of cytochrome *c* oxidase activity in porcine brain with NIR

**Fig. 2:**
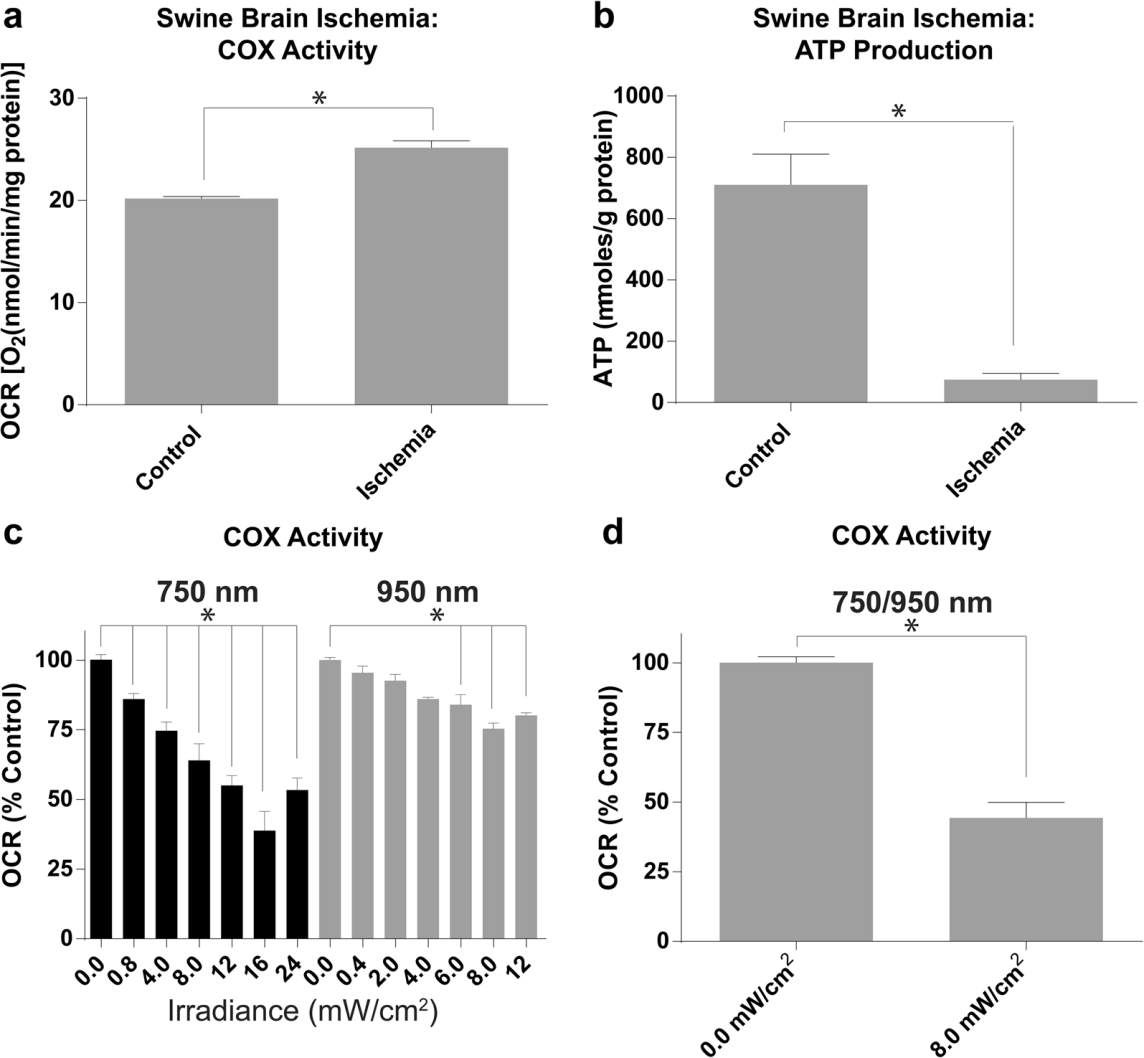
NIR modulates COX function. **A** COX activity and **B** ATP levels in control and ischemic porcine brain. Effect of NIR on isolated brain COX using **C** 750 and 950 nm alone and **D** in combination. The oxygen consumption rate was analyzed in the absence of NIR (0.0 mW/cm^2^), or presence of NIR at the indicated power density, applying 750 nm (C—black) and 950 nm alone (C—gray) or the combination (D – 750 at 8 mW/cm^2^, 950 at 4 mW/cm^2^). Data represented as Mean + SD

To analyze the effect of NIR on porcine COX, we purified the enzyme and analyzed enzyme kinetics with an Oxygraph O_2_ electrode. ATP levels were reduced in the ischemic porcine brain (Fig. 2A) and COX activity was increased (Fig. 2B). The effect of 750 nm and 950 nm NIR on COX activity was recorded alone and in combination. Both 750 nm and 950 nm NIR significantly reduce the respiratory activity of COX (Fig. 2C). The inhibitory effect becomes saturated at 61% for 750 nm and 25% for 950 nm (i.e. oxygen consumption reduced to a maximum of 39% and 75% of control respectively). Combined application of 750 nm and 950 nm NIR showed significant inhibition (56%) (Fig. 2D). In these isolated enzyme experiments, COX is solubilized and not bound to the inner mitochondrial membrane and therefore the absolute intensities necessary to inhibit COX in vitro do not directly represent the dose necessary for a therapeutic effect in vivo. These data are nonetheless important, as they provide evidence for inhibition of COX by these two NIR wavelengths and demonstrate a point of saturation for each wavelength.

### Prevention of early mitochondrial swelling and mitophagy following ischemia/reperfusion injury with NIR

**Fig. 3:**
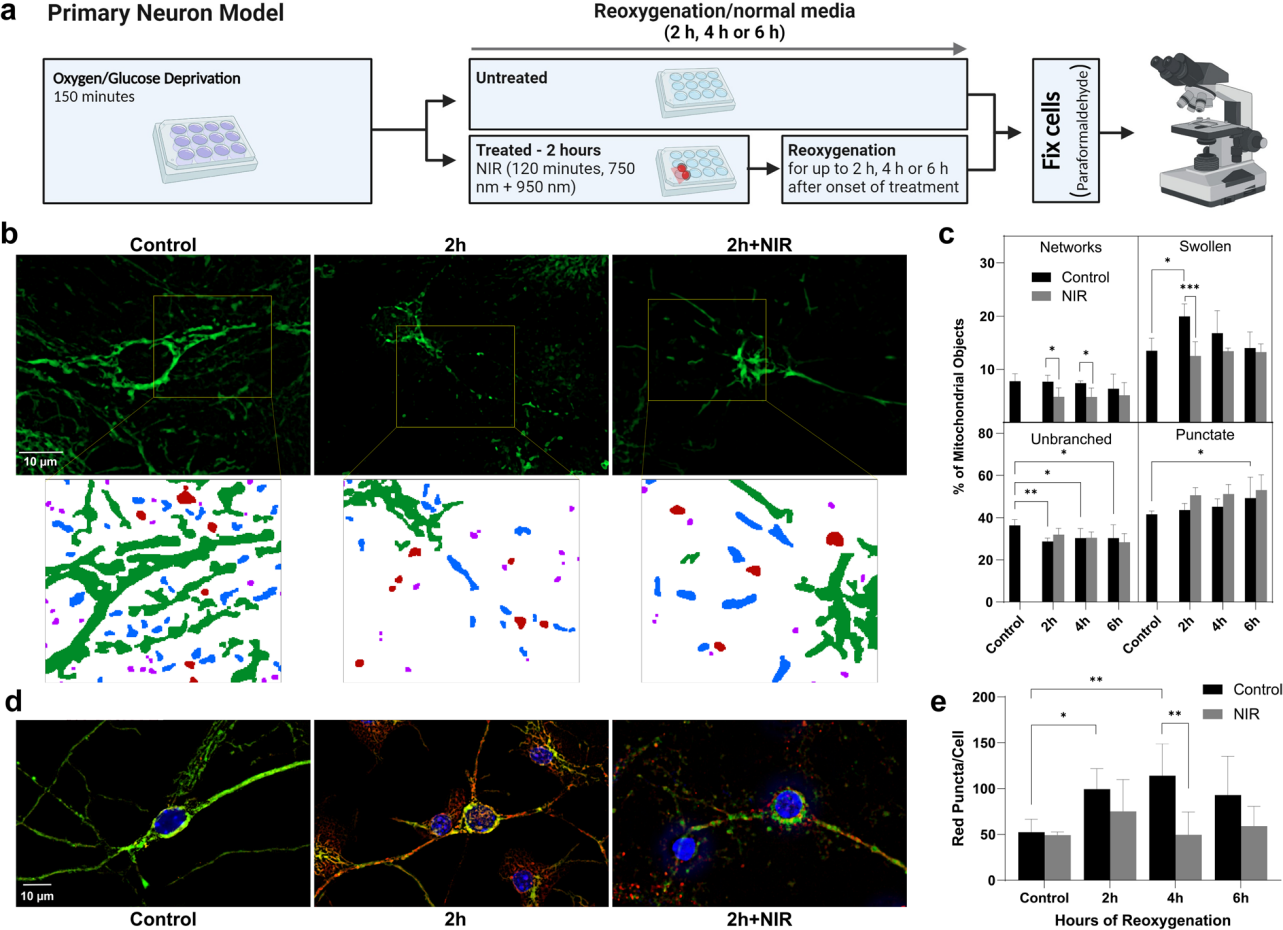
NIR limits post-ischemic mitochondrial swelling and reduces mitophagy in primary neurons. **A** Experimental approach **B** Representative images of MitoQC (GFP) for machine learning classification. Inserts: binary maps of mitochondrial objects labeled by morphological classification (networks: green, unbranched: blue, red: swollen, magenta: punctate). **C** Mitochondrial network analysis following OGD + 2 h, 4 h and 6 h of reoxygenation. Mitochondrial network analysis with and without NIR therapy. (n = 6 per group, *p < 0.05, **p < .01, ***p < 0.001, by two-way ANOVA and post hoc Tukey test, mean + SD). **D** Primary neurons extracted from MitoQC mice show increased mitophagy following OGD (red signal). **E** Mitophagy is significantly reduced with NIR therapy. (*n* = 5 per group, *p < 0.05, **p < 0.01, by two-way ANOVA post hoc Tukey, data represented as Mean + SD)

Modulation of mitochondrial function is hypothesized to reduce mitochondrial damage and downstream induction of cell death mechanisms and/or mitochondrial turnover through mitophagy. Mitochondrial morphologic disruption in primary mouse neurons was assessed through GFP imaging in mitoQC mice to identify mitochondria (Fig. 3B). Our analysis demonstrates that mitochondrial morphology is disrupted after OGD, with an increase in mitochondrial fragmentation. We observed a significant reduction in unbranched mitochondria from 36% in control cells to 29%, 30% and 30% at 2 h, 4 h and 6 h of reoxygenation, respectively, following OGD and a significant increase in punctate phenotype from 42% in control cells to 49% at 6 h after OGD. We also report a rapid and significant transition to a swollen morphology from 13% of mitochondria in control cells to 20% at 2 h of reoxygenation after OGD (Fig. 3B and C). Importantly, studies with NIR treatment demonstrate no effect on mitochondrial fragmentation, but a significant reduction in mitochondrial swelling, a morphology causally linked to mechanisms of neuronal death, with a ~ 35% reduction in the prevalence of swollen mitochondria (20% swollen mitochondria at 2 h reoxygenation in control cells vs. 13% swollen mitochondria after NIR treatment) (n = 6 per group, by two-way ANOVA and post hoc Tukey test).

Maintenance of mitochondrial integrity is critical to preserve cellular function and prevent post-ischemic cell death. Our previous studies suggest that mitophagic flux is significantly upregulated in neurons destined to die following ischemia, and we propose that mitochondrial damage induced by I/R leads to an unsustainable induction of this recovery pathway [48]. Our data presented here demonstrate a significant increase in red puncta per cell following 2 and 4 h of reoxygenation, which indicates mitochondrial localization to lysosomes (GFP-negative, mCherry-positive mitochondrial objects, *n* = 5 per group, by two-way ANOVA post hoc Tukey). Importantly, mitoQC neurons exposed to OGD with NIR treatment have significantly reduced red puncta mitochondrial objects, suggesting that NIR mitigates mitophagy (Fig. 3D, gray bars), which we propose is a downstream consequence of limiting overall mitochondrial injury during reperfusion post-resuscitation treatment with NIR reduces neuronal loss in the CA1/3 hippocampus and improves neurologic function scores in a clinically relevant porcine model.

Ischemia and reperfusion cause neurological injury and contribute to death and disability following cardiac arrest. Accordingly, the pivotal translational aims of this study were to: (1) establish that NIR can be safely delivered to large subjects with adequate depth of penetration to reach brain regions affected by I/R injury, and (2) evaluate the neuroprotective efficacy of partial COX inhibition with NIR in a clinically relevant large animal model of cardiac arrest.

**Fig. 4:**
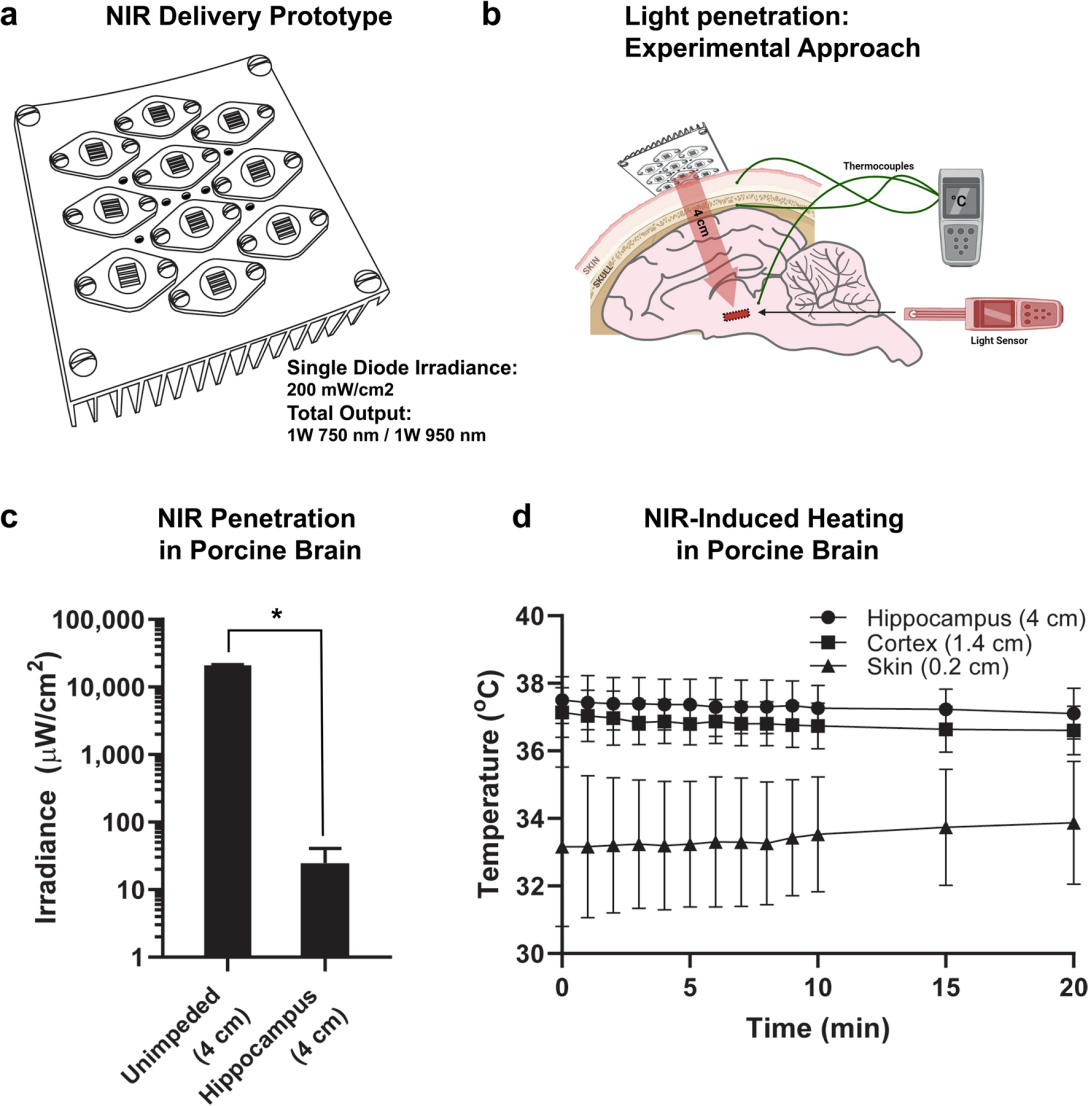
Brain tissue penetration and temperature. **A** Diagram of the NIR treatment device with light emitting diode arrays fixed to an aluminum heat sink. **B** Diagram of the surgical implantation of photodiode and thermocouple. **C** NIR penetration measurements, measured with an implanted photodiode at 4 cm performed through the swine head, measured at the hippocampus compared to unimpeded measured at 4 cm distance from the LEDs (i.e., without tissue between the LED and the power meter. **D** Temperature was measured during NIR treatment with thermocouples placed in the skin (0.2 cm), surgically implanted in the surface of the cortex (1.5 cm), and the hippocampus (4 cm). Data represented as Mean + SD

We quantified the penetration of NIR to brain regions significantly injured by global brain ischemia (hippocampus) by measuring the light loss through the scalp/skull/brain in anesthetized subjects. NIR was applied from the LED-treatment prototype (Fig. 4A) positioned on the superior surface of the head, and a NIR meter was positioned at the level of the hippocampus, as illustrated in Fig. 4B (4 cm depth). Light attenuation was calculated by comparing light transmission from the scalp to the implanted photodiode (hippocampus–4 cm, *n* = 3) and comparing to light transmission through air at the same distance (unimpeded–4 cm, *n* = 4). Light transmission averaged 0.12% of the unimpeded light levels through the porcine brain (Fig. 4C). To deliver NIR safely, it is imperative to eliminate tissue heating. In this regard, no significant changes in brain temperature at the cortical surface or hippocampus were observed during NIR therapy compared to T0 with the NIR prototype device (n = 3, 2-way ANOVA with repeated measures, p = NS over time; Fig. 4D).

**Fig. 5:**
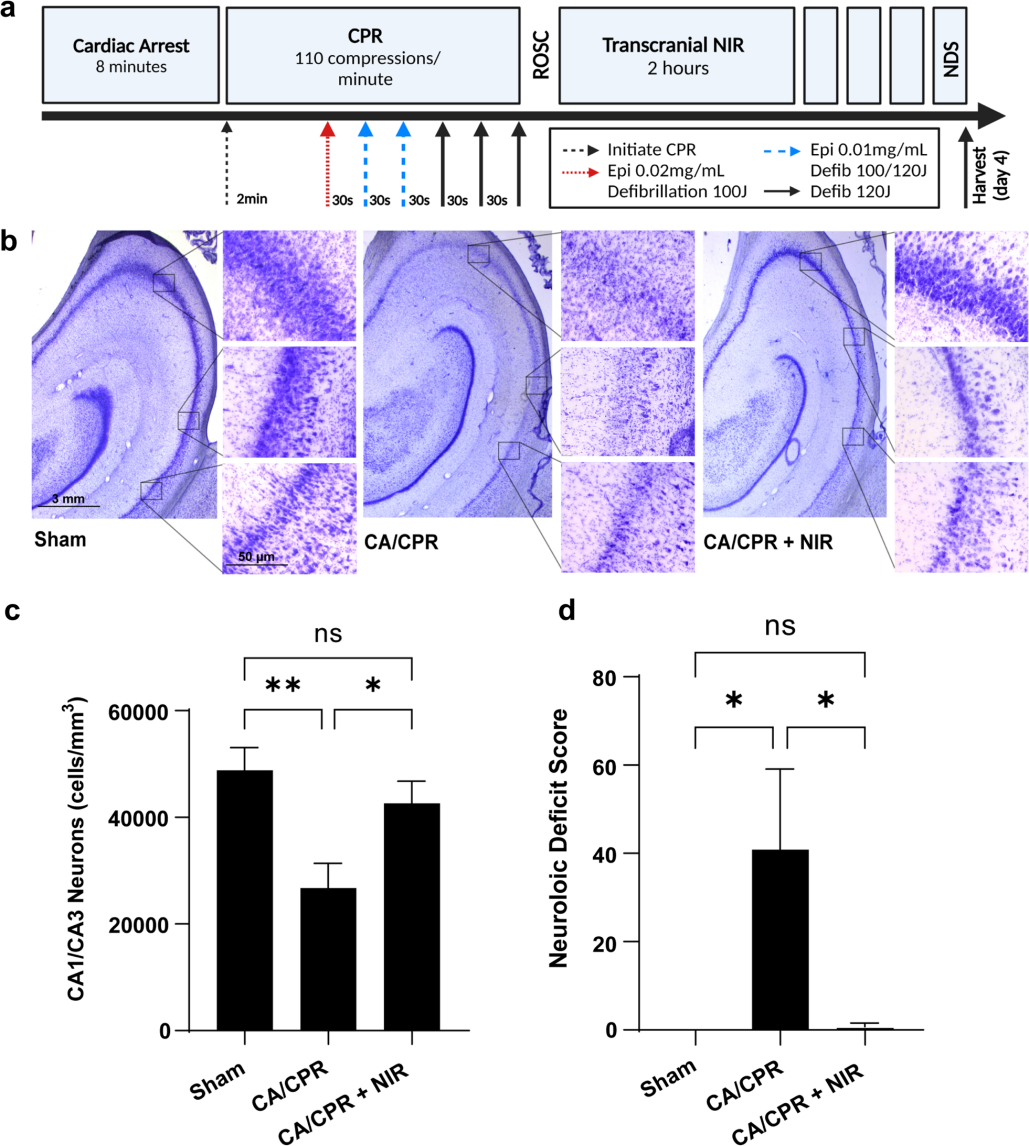
Neuroprotective effect of NIR. **A** The experimental protocol for cardiac arrest, resuscitation and recovery in porcine. **B** Micrographs showing cresyl violet staining of the hippocampus (2.5× magnification) and random regions in the CA1 and CA3 hippocampus (inset, 20× magnification). **C** Stereological quantification of remaining neurons in the CA1/CA3 regions of the hippocampus. The sum of neurons and tissue volume were quantified in the hippocampus bilaterally to acquire the density of neurons (cells/mm^3^). **D** Neurologic deficit score was recorded four days after arrest. Sham, *n* = 10; CA/CPR, *n* = 11 (2 did not achieve ROSC) and CA/CPR + NIR, *n* = 9. The mean number of neurons and neurologic severity score for each group are represented and were compared with Newman–Keuls multiple comparison test, *p < 0.05, **p < 0.01

Cardiac arrest was induced in a separate cohort of animals (sham, n = 10; CA/CPR, n = 11, 2 did not achieve ROSC; and CA/CPR + NIR, n = 9) for 8 min by delivering a direct current pulse to the myocardium with a pacing wire. Ventricular fibrillation was left untreated for 8 min, followed by CPR with continuous chest compressions (Fig. 5A). Cardioversion and epinephrine were administered until ROSC, then transcranial NIR was delivered with our prototype LED delivery device (Fig. 4A) delivering a total of 1W 750 nm and 1W 950 nm for 2 h post-ROSC. A hallmark of cardiac arrest and resuscitation is neuronal cell death in the CA1/CA3 region of the hippocampus with attendant neurological deficits (Fig. 5B & C). Therefore, primary outcome measures used to evaluate neuroprotection were neurologic deficit scores at 4 days and histologic assessment of neuronal loss in the CA1/CA3 region of the hippocampus. We found that eight minutes of cardiac arrest followed by cardiopulmonary resuscitation (CA/CPR) resulted in a 45% reduction in neuronal density in the hippocampus at 4 days post-ROSC when compared with sham-operated subjects (26,743 ± 4651 vs. 48,843 ± 4208, *p* < 0.05; Fig. 5B). Two-hour treatment with 750 nm + 950 nm NIR evoked significant neuroprotection, enhancing neuronal survival by ~ two-fold when compared with untreated CA/CPR (42,612 ± 4176 vs 26,743 ± 4651, *p* < 0.05; Fig. 5B). Neurological recovery is the cornerstone for the assessment of patient recovery following cardiac arrest. We found cardiac arrest causes marked increases in neurologic deficit scores compared to shams (40.8 ± 18.3 vs. 0.0 ± 0.0; Fig. 5C), while treatment with NIR was accompanied by a near-complete absence of neurologic deficits (score of 0.9 ± 0.9, *p* < 0.05 versus CA/CPR; p = ns versus sham; Fig. 5C). Importantly, the favorable effects of NIR treatment could not be attributed to differences in arrest severity and CPR quality: end-tidal CO_2_ during CPR and the time to ROSC did not differ among groups. Furthermore, while blood gasses (pH, PCO_2_, spO_2_, HCO_3_^−^) or blood lactate concentration prior to arrest and 30 min after ROSC were significantly different within groups, there were no across-group differences (i.e., NIR treatment vs. placebo) in blood gasses, blood lactate or etCO_2_ during CPR (Table 1, mixed effects analysis with repeated measure, multiple comparisons with Sidak’s test). This study was not powered to detect sex specific differences, however no apparent sex specific differences were detected in outcomes and both sexes had numerically higher average neuron counts in NIR treated versus CA/CPR untreated animals (sex stratified neuron counts: Sham–male 45,062 ± 16,228 vs female 54,515 ± 4575; CA/CPR–male 27,025 ± 12,626 vs female 26,648 ± 14,503; CA/CPR + NIR – male 38,459 ± 6503 vs female 47,803 ± 17,247).

## Discussion

Neurological injury is the leading cause of death and disability following cardiac arrest and, because treatment options are limited, the clinical outcomes remain poor [49]. In this study, we demonstrate robust neuroprotection with a novel and noninvasive mechanism-based technology in a translational, clinically relevant model of cardiac arrest and resuscitation.

### Therapeutic NIR: biological mechanisms of protection

The biological effect of therapeutic NIR is attributed, at least in part, to modulation of mitochondrial function. More specifically, metal centers in COX broadly absorb NIR, and it was previously proposed that this causes activation of enzymatic function and, consequently, stimulation of mitochondrial respiration [50–56]. By this reasoning, early hypotheses proposed that NIR would improve outcomes by increasing ATP availability. The therapeutic effect of NIR has been documented in models of skeletal muscle ischemia, in myocardial infarction and cerebral I/R and in clinical studies of ischemic stroke [26–29, 31–36, 57–59]. In contrast, our group discovered characteristics of NIR using a novel approach by scanning light in the near-infrared spectrum for effects on COX activity [39]. We observed that, in addition to stimulatory NIR (810 nm), distinct wavelengths (750 nm and 950 nm) have an inhibitory effect on COX activity and mitochondrial respiration. Consistent with evidence using stimulatory wavelengths, we demonstrated that the inhibitory effect is transient and fully reversible, and respiration recovers rapidly after NIR exposure is suspended [39]. In support of our previous findings, we report a dose-dependent and saturable inhibition of isolated COX with 750 nm and 950 nm light. We proposed that inhibitory wavelengths of NIR would prevent hyperpolarization of the ΔΨ_m_ and thus generation of cytotoxic ROS, which are generated at pathologically high ΔΨ_m_ levels during the early phase of reperfusion [19, 60]. Consistent with this concept, we have reported that inhibitory wavelengths of NIR reduced ROS generation in transient global brain ischemia in the rat, which was accompanied by attenuated neurological injury and improved neurocognitive assessment [39]. It should be noted that even a small reduction of ΔΨ_m_ during reperfusion due to partial inhibition of COX activity can, as shown here, translate into a profound neuroprotective outcome. We posit that this is because the correlation of the magnitude of the mitochondrial membrane potential and the amount of ROS produced by the mitochondria is of exponential nature. Furthermore, lower NIR energy densities at the deeper hippocampal brain structures are still expected to lead to significant reduction of ΔΨ_m_ because measurements done with purified and detergent-solubilized COX do not consider ΔΨ_m_; i.e., purified COX does not have to pump protons against a proton gradient present in intact cells, which is the main inhibitor of the proton pumps under in vivo conditions, classically referred to as respiratory control. We thus propose that NIR power densities in the double digit µW/cm^`^ range as quantified here and in our cadaver studies are sufficient to limit ΔΨ_m_ hyperpolarization, ROS production and cell death [61, 62]. The data presented here demonstrate that reducing COX activity with NIR therapy during reperfusion also limits swelling of mitochondria during reperfusion and limits the induction of mitophagy, two mechanisms central to the progression of mitochondrial failure and cell death during reperfusion [48].

Importantly, this mechanism does not conflict with the established concept that NIR can stimulate mitochondrial respiration, but rather contributes to an understanding of the bioactive and wavelength-specific characteristics of NIR. Preclinical studies of cerebral ischemia have demonstrated that stimulatory wavelengths, 808 nm and 610 nm, provide reparative and healing properties through delayed and repeated treatments [30–33, 63–66], potentially mediated via inflammatory modulation, release of signaling molecules and even induction of angiogenesis and neurogenesis [30, 67–69]. Alternatively, our studies indicate that early treatment with inhibitory wavelengths prevent generation of ROS during reperfusion to provide neuroprotection [39, 70]. These data suggest that: (1) the biological effect of NIR is wavelength-dependent; and (2) both stimulatory and inhibitory wavelengths may have salutary effects, depending on when and where treatment is applied. Further investigation is required to evaluate the additive effect of targeting stimulatory and inhibitory wavelengths to specific injury phases.

### Identifying therapeutic thresholds for therapeutic NIR intensity

The biological effect of NIR is dependent on dose, which is typically determined by the light intensity and time. More specifically, evidence suggests that NIR has an optimal irradiance that evokes a maximal effect [71, 72]. The majority of these studies use cellular responses and therapeutic outcomes to measure the effect of NIR [73]. To our knowledge, ours are the first studies that interrogate the real-time effect of NIR intensity on COX activity. Consistent with the established paradigm, we demonstrate that the effect of NIR on COX is dose dependent and saturable (Fig. 2). Importantly, the NIR energy densities determined in isolated COX studies are not directly applicable to determining a dosing strategy for use in vivo. Measurements done with purified and detergent-solubilized COX do not consider ΔΨ_m_; i.e., purified COX does not have to pump protons against the proton gradient that is present in intact mitochondria, which is the main inhibitor of the COX under in vivo conditions, classically referred to as respiratory control.

### Translational development for noninvasive transcranial mitochondrial modulation

Translational development is a critical and, to date, largely elusive step toward clinical application of treatments for acute cerebral injury. Many therapeutic strategies have advanced to clinical trials, though few are approved for use in humans [1, 74, 75]. Currently, hypothermic targeted temperature management (HTTM) is the only treatment that has demonstrated clinical efficacy in cardiac arrest patients, however recent multi-center randomized clinical trials have brought into question the efficacy of HTTM compared to targeted temperature management alone [76–78]. The barriers that limit clinical translation of experimental treatments are unclear; however, developing neuroprotective technology in clinically relevant models is crucial. Accordingly, the primary objective of this study was to build on our mechanistic foundation and preclinical groundwork to develop therapeutic NIR toward clinical use [39].

Histopathological and neurocognitive evidence from this study demonstrate that transcranial 750 nm and 950 nm NIR administered after ROSC improves cardiac arrest-induced brain injury in the translationally relevant large animal model. Compelling evidence from our lab and others has previously reported neuroprotection with NIR in models of forebrain ischemia–reperfusion and focal ischemic stroke [30, 32, 33, 39, 70]. However, this is the first study to evaluate transcranial NIR as a treatment for acute brain injury in a large animal model. Our study design addresses two important aspects that facilitate translational development; (1) the porcine model of cardiac arrest causes a clinically relevant brain injury by induced ventricular fibrillation with circulatory arrest followed by standard resuscitation measures (CPR); (2) the porcine anatomy is analogous to human which is critical to evaluate the feasibility of treating vulnerable brain structures with therapeutic NIR. In this study, we demonstrate that transcranial NIR reaches a therapeutic dose in the hippocampus (located ~ 4 cm of tissue depth in pigs) which supports previous studies by our group and others that report effective penetration into human cadaver brains [61, 62, 79, 80] as well as clinical evidence of efficacy in neurological conditions such as dementia, depression, Alzheimer’s disease and Parkinson’s disease [81, 82]. While the hippocampal neurons of the CA1/CA3 are highly vulnerable to ischemia, other brain structures may be subjected to injury. Due to scattering, the use of light emitting diodes which produce non-collimated light, and the surface area of irradiation, NIR is likely to reach most of the forebrain. We did not quantify injury in other vulnerable regions of the brain, however we predict that NIR treatment provides impartial neuroprotection against cellular I/R injury. Indeed, salvage of neurons throughout the brain, in addition to the hippocampus, is likely to have contributed to the observed improvement in deficit severity.

This study has important limitations. Although porcine cardiac arrest is well-established and clinically relevant, there are weaknesses related to this model; (1) in contrast to the patient population, the subjects studied were young and healthy; (2) This model causes moderate brain injury, compared to the more severe injuries associated with out-of-hospital cardiac arrest in patients; (3) The porcine brain is smaller than that of adult humans, which allows greater NIR penetration to vulnerable structures. It is also important to consider that experimental animals were 3–4 months old and are considered juvenile which limits the relevance of our model to the adult and elderly cardiac arrest population. Although this is a commonly used model, the efficacy of NIR will need to be elucidated in adult and senescence models [83]. Another limitation is that we did not interrogate NIR dose (irradiance, treatment duration) or delayed application. Future studies will be necessary to evaluate neuroprotection in models of adult cardiac arrest, in the context of comorbidities and in more severe cardiac arrest and to investigate alternative treatment paradigms that optimize therapeutic potential in humans. These limitations should be considered during clinical trial design.

## Conclusions

This study supports our novel finding that COX-inhibitory NIR can evoke neuroprotection in cardiac arrest. We report that therapeutic NIR can be used in a clinically relevant model to noninvasively mitigate brain injury and improve neurocognitive recovery following cardiac arrest. To our knowledge, this is the first evidence demonstrating efficacy of transcranial NIR in a large animal system which supports feasibility in human subjects. Lastly, we provide a framework for scaling treatment parameters in future investigations in other models and in clinical studies.

### Supplementary Information

**Additional file 1: Fig. S1.** Stereology method. A coronal slice of the hippocampus stained with cresyl violet. The pyramidal neuronal layer was traced to include the CA1 and CA3, shown outlined in red. Regions of interest were randomly selected by the stereology software and neurons were counted at 63x within the region of interest.

**Additional file 2: Table S1.** Neurological deficit score. Neurologic deficits were assessed using a scoring system that evaluated consciousness, brainstem function, sensory responses, motor function, postural reflexes, mobility, spatial orientation, activity, and seizures four days after surgery.

## Data Availability

We will share data that include values and statistical analysis associated with this manuscript upon reasonable request. This study utilizes machine learning analysis developed previously, which is available in the cited article. There is no sequencing or proteomic, or other type of bioinformatics/population data included in the manuscript. Furthermore, there are no data generated from human subjects. The corresponding author will oversee execution of data management and sharing.
