# Supplementary figures and images for: Modulation of mitochondrial function with near-infrared light reduces brain injury in a translational model of cardiac arrest
Source: Crit Care. 2023 Dec 14;27:491. doi: 10.1186/s13054-023-04745-7 (PMC10720207; doi:10.1186/s13054-023-04745-7)

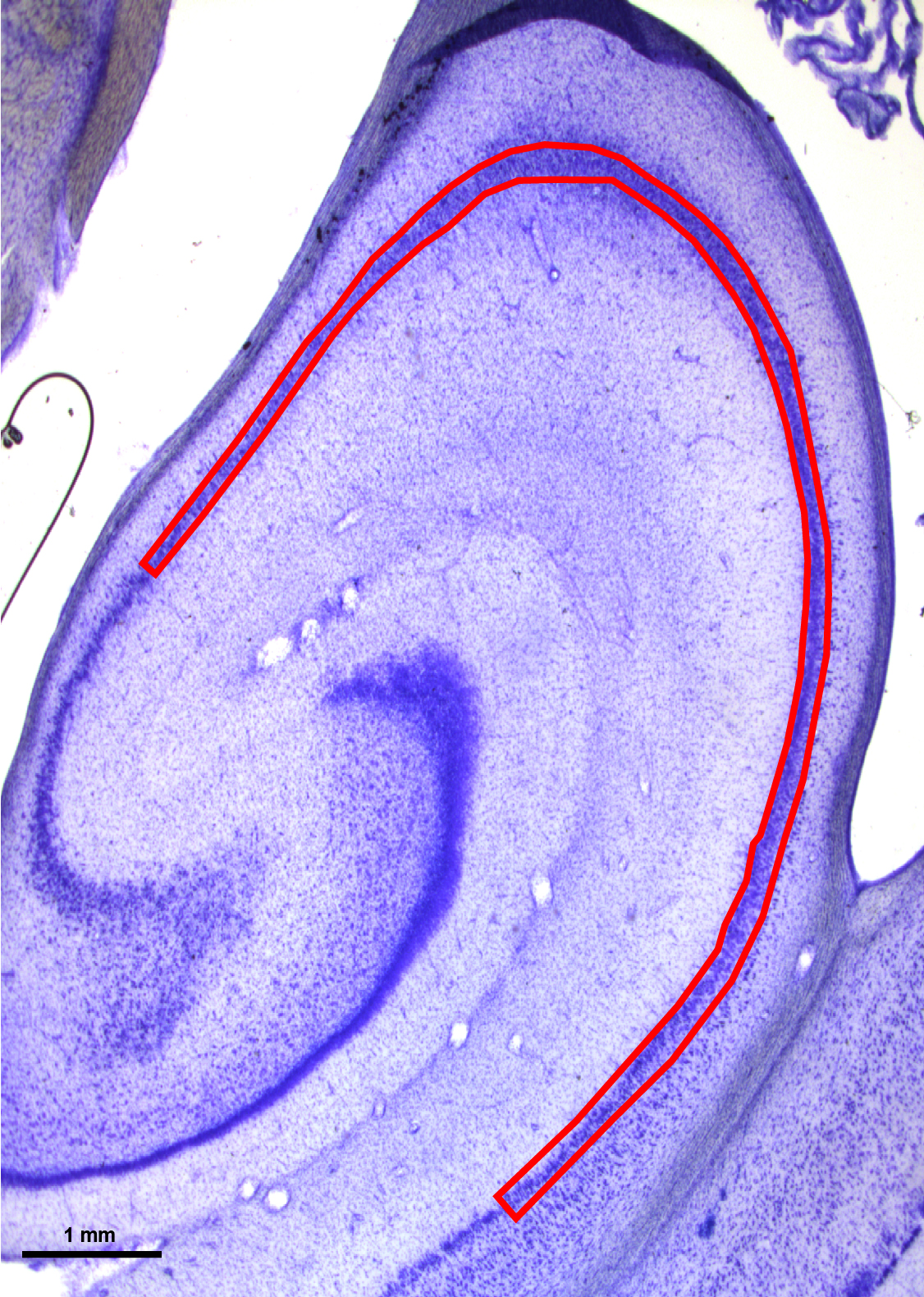

Supplement: Supplementary file 1 — Additional file 1: Fig. S1. Stereology method. A coronal slice of the hippocampus stained with cresyl violet. The pyramidal neuronal layer was traced to include the CA1 and CA3, shown outlined in red. Regions of interest were randomly selected by the stereology software and neurons were counted at 63x within the region of interest. [file 13054_2023_4745_MOESM1_ESM.jpg]
